# An integrated TMS-EEG and MRI approach to explore the interregional connectivity of the default mode network

**DOI:** 10.1007/s00429-022-02453-6

**Published:** 2022-02-04

**Authors:** Romina Esposito, Marta Bortoletto, Domenico Zacà, Paolo Avesani, Carlo Miniussi

**Affiliations:** 1 Center for Mind/Brain Sciences–CIMeC, University of Trento, Corso Bettini 31, 38068 Rovereto, TN Italy; 2 IRCCS Istituto Centro San Giovanni di Dio Fatebenefratelli, via Pilastroni 4, 25125 Brescia, Italy; 3 Neuroinformatics Laboratory, Center for Information Technology, Fondazione Bruno Kessler, via Sommarive 18, 38123 Trento, Italy; 4 Centre for Medical Sciences, CISMed University of Trento, Via S. Maria Maddalena 1, 38122 Trento, Italy

**Keywords:** Integrative neuroscience, Connectivity, MRI, TMS-EEG, Tractography, Default mode network

## Abstract

Explorations of the relation between brain anatomy and functional connections in the brain are crucial for shedding more light on network connectivity that sustains brain communication. In this study, by means of an integrative approach, we examined both the structural and functional connections of the default mode network (DMN) in a group of sixteen healthy subjects. For each subject, the DMN was extracted from the structural and functional resonance imaging data; the areas that were part of the DMN were defined as the regions of interest. Then, the target network was structurally explored by diffusion-weighted imaging, tested by neurophysiological means, and retested by means of concurrent transcranial magnetic stimulation and electroencephalography (TMS-EEG). A series of correlational analyses were performed to explore the relationship between the amplitude of early-latency TMS-evoked potentials and the indexes of structural connectivity (weighted number of fibres and fractional anisotropy). Stimulation of the left or right parietal nodes of the DMN-induced activation in the contralateral parietal and frontocentral electrodes within 60 ms; this activation correlated with fractional anisotropy measures of the corpus callosum. These results showed that distant secondary activations after target stimulation can be predicted based on the target’s anatomical connections. Interestingly, structural features of the corpus callosum predicted the activation of the directly connected nodes, i.e., parietal-parietal nodes, and of the broader DMN network, i.e., parietal-frontal nodes, as identified with functional magnetic resonance imaging. Our results suggested that the proposed integrated approach would allow us to describe the contributory causal relationship between structural connectivity and functional connectivity of the DMN.

**Supplementary Information:**

The online version contains supplementary material available at 10.1007/s00429-022-02453-6.

## Introduction

In recent decades, researchers have expressed renewed interest in exploring brain dynamics by shifting their attention from local activations to brain connectivity. One of the reasons for this change in focus is that the pattern of connections that are activated in relation to an area and a task, rather than the sole activation of the area itself (network "segregation" and "integration"; Sporns 2013), is now thought to have an important role in sustaining the brain capability. The brain is persistently active to ensure that different specific patterns based on the state or task demands are provided. Even the resting state is a well-defined "operational" brain state, which corresponds to a specific network activity pattern (Raichle 2015). In this regard, accumulating evidence points to a large number of brain regions, for instance, the default mode network (DMN), that are functionally connected even at rest (Greicius et al. 2009; Raichle 2015). Therefore, even in the absence of task-related neuronal activation and “specific” external input, all these synchronized brain regions form resting-state networks (van den Heuvel and Hulshoff Pol 2010). This high level of functional connectivity between regions suggests the existence of a distinctive brain structural architecture to facilitate such ongoing interregional communication (Boorman et al. 2007; Bortoletto et al. 2021; Momi et al. 2021; Quentin et al. 2015; Silverstein et al. 2020). Thus, understanding how to measure such dynamic patterns underlying brain functions is one of the crucial questions in neuroscience.

In this work, we used an innovative approach to study the relationship between structural connectivity and functional connectivity in the DMN. The DMN was selected for this investigation because it is a well-defined network, and it can be tested while a person is in the resting state. In detail, the focus of this study was on the relationship between structural architecture and the dynamics of the main cortical nodes of the DMN. The DMN has been proposed to play a crucial role in core processes of human cognition (Raichle et al. 2001; Raichle 2015), including mind wandering, goal-directed behaviour, and relating oneself to the outside world. Therefore, the DMN is an ideal target to study to reach a more detailed understanding of the relation between structure and function and of DMN connectivity as a whole.

The majority of studies on brain connectivity have been obtained from stand-alone unimodal neuroimaging methods (Plis et al. 2011). We proposed the use of an integrative approach with a combination of different methods (Bergmann et al. 2016) composed of structural and functional magnetic resonance imaging (MRI and fMRI, respectively), diffusion-weighted imaging (DWI), and transcranial magnetic stimulation and electroencephalography (TMS-EEG) coregistration (Bortoletto et al. 2021; Esposito et al. 2020; Levy-Lamdan et al. 2020; Momi et al. 2021; Voineskos et al. 2010).

Our project was initiated with the idea that with the proposed integrative approach combining TMS-EEG guided by MRI, it would be feasible to obtain a detailed view of the spatial (MRI reveals the structural pathways), temporal (EEG enables measurements of the time course of the activity of the cortical areas) and effective (TMS provides information about directionality) features of the DMN at a macroscopic level. The proposed methodologically integrated MRI-TMS-EEG approach represents a tool that can be used to describe the relationship among the structural, functional, and effective connectivity of brain networks (Voineskos et al. 2010). The farsighted contribution of the integrated approach, applied in this experiment, consists of reflecting both the spatial organization and the temporal dynamics of the DMN (Momi et al. 2021). This research aspires to validate a method that allow us to increase our understanding of effective neural interactions in the brain.

In the recent literature, the functional nodes that compose the DMN are the precuneus, bilateral medial frontal regions, and bilateral inferior parietal regions. To pursue the aim of this study, only cortical nodes of the DMN accessible by TMS (i.e., the bilateral medial prefrontal and inferior parietal regions) were tested. We acquired MRI and fMRI data when individuals were in the resting state; subsequently, we computed the structural pathways between the regions in the DMN network using the DWI data. Finally, DMN cortical excitability and effective connectivity were assessed with TMS-EEG coregistration guided by MRI data (i.e., neuronavigation). The combination of single-pulse TMS and EEG recording created the opportunity to directly investigate the contributory causality of the stimulated node within networks (Bortoletto et al. 2015).

For each participant, we were able to identify the functional DMN via fMRI data, so it was possible to precisely find the coordinates of the nodes to stimulate during the TMS-EEG recording sessions. Moreover, these coordinates were used to keep the position of the TMS coil during registration to ensure the stimulation of the same cortical node within the sessions. Notably, functional regions of interest (ROIs) have also been used to define the structural pathways underlying the functional network. The investigation of all the functional and structural measurements was performed at the individual level, resulting in a high constancy distribution among the samples. The comparison of the two TMS-EEG sessions also contributed to confirm efficacy of the proposed method in measuring a stable response over time. The session comparison was important evidence of the efficacy and, therefore, replicability of the measurements of the proposed approach. Moreover, from the integrated MRI, TMS, and EEG approach, it was possible to obtain outcomes for the temporal dynamics of signal distribution through the correlation of the TMS-evoked potentials (TEPs) and fractional anisotropy (FA).

## Materials and methods

### Experimental design and participants

**Fig. 1:**
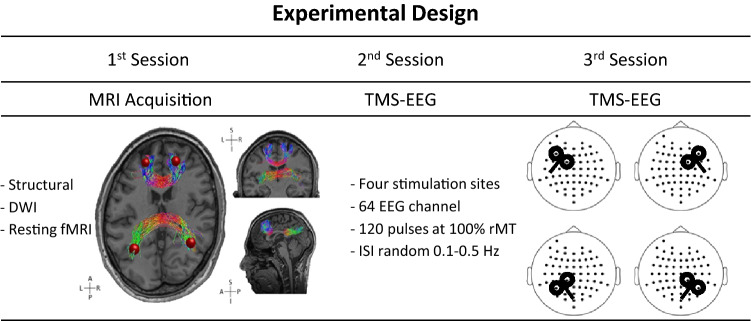
Main characteristics of the experimental design, which is composed of three sessions. The first session entails MRI acquisition (structural, DWI, functional MRI). The second and third sessions comprised two identical TMS-EEG recording procedures. The lower part of the figure represents the structural MRI of one subject upon which the functional ROIs (red spheres) connected by the relative direct structural connections and the TMS coil orientation for the four stimulation conditions/areas are overlaid

Thirty-five subjects were enrolled and screened for their compatibility with MRI and TMS-EEG equipment through a screening questionnaire (Rossi et al. 2009; Sammet 2016). All subjects were right-handed and reported no history of neurological deficits. The study was composed of three sessions (Fig. 1), one for MRI acquisition, and approximately 10 months later (mean days 290.6 ± 42.4), the first of the two TMS-EEG sessions was recorded (see below for details). Even though the gap between the MRI and the two TMS-EEG sessions was quite long, there is evidence that the reliability of the resting-state networks, over time, is higher than that of other brain networks (see Noble et al. 2019). For the second and third sessions, the sample was reduced to a total of 18 and 16, respectively, due to subjects dropping out or technical problems. Therefore, the final sample, which underwent all the testing sessions, was composed of 16 young subjects (9 males, mean age 25.2 ± 2.2 years; 7 females, mean age 23.6 ± 3.3 years).

During the MRI session, structural, resting-state functional, and DWI data were recorded for each subject. During the acquisition of the imaging data, subjects were asked to lay still with their eyes open, while a white fixation cross was projected on the screen. The two TMS-EEG sessions were performed in an identical manner 2 months apart on average (mean days 72.3 ± 35.8). During the TMS-EEG sessions, TMS was applied over four different areas of the brain, the right and left prefrontal and parietal areas, while EEG was recorded concurrently (see below for details). For each subject, each TMS-EEG session was divided into six blocks: two 5-min adaptation blocks of resting EEG recorded at the beginning and the end of the session (data not analysed) and four TMS-EEG blocks. Subjects wore earplugs, kept their eyes open, and were seated in a comfortable armchair in an acoustically isolated room. In all blocks, participants were instructed to fixate on a white fixation cross on a computer screen at a distance of 70 cm. The stimulation blocks were interleaved by 5-min breaks, and participants were allowed to move during that time. During breaks, a short video was presented on the computer screen. These videos, which consisted of short parts of a documentary about the planet and universe, were intended to attract the subject's attention and enable a distinction between a state of cognitive involvement and a resting state. EEG signals were not recorded during these periods. The current study was approved by the Human Research Ethics Committee of the University of Trento. Written informed consent was obtained from all subjects before they became involved in the study. All safety MRI and TMS procedures and guidelines were respected (Rossi et al. 2009; Sammet 2016). Brain Imaging Data Structure (BIDS)-formatted data from this study are available on gin.g-node.org/CIMeC/TMS-EEG_brain_connectivity_BIDS.

### MRI

MRI data were acquired at the Center for Mind/Brain Sciences (CIMeC—University of Trento, Italy) using a 4 T MRI system equipped with an 8-channel receive head RF coil (Bruker MedSpec Synco). Functional images were acquired with a single shot T2*-weighted gradient-recalled echo-planar imaging (EPI) sequence. A 30-slice protocol was employed, acquiring images in ascending interleaved order, within a repetition time (TR; the amount of time between successive pulse sequences applied to the same slice) of 2000 ms [voxel resolution, 3 × 3x3 mm^3^; echo time (TE), 28 ms; flip angle, 73°; field of view (FOV), 192 × 192 mm]. Two hundred consecutive brain volumes were acquired. To coregister the low-resolution functional images to a high-resolution anatomical scan, we acquired two T1-weighted anatomical scans [magnetization-prepared rapid gradient-echo (MP-RAGE) 1 × 1 × 1 mm^3^; FOV = 256 mm; 176 slices; generalized autocalibrating partially parallel acquisition (GRAPPA) with an acceleration factor of 2; TR = 2700/2500 ms; TE = 4.18/3.37 ms; inversion time (TI) = 1020/1200 ms; flip angle = 7°/12°]. For whole-brain tractography, the DWI scheme was acquired using a spin echo EPI sequence (TR = 7100 ms, TE = 99 ms) with a b-value of 1500 s/mm2. Ten volumes without any diffusion weighting (b0-images) and 30 diffusion-weighted volumes (isometric voxel 2.3 × 2.3 × 2.3 mm^3^) were acquired.

### EEG

EEG signals were acquired using a TMS-compatible EEG system (BrainAmp DC Brain Products GmbH, Germany), with continuous recording from 61 scalp electrodes (FCz, FP1, FP2, AF7, AF8, F7, F5, AF3, AFz, AF4, F8, F6, F3, F1, Fz, F2, F4, FT7, FC5, FC3, FC1, FC2, FC4, FC6, FT8, T7, C5, C3, C1, Cz, C2, C4, C6, T8, Tp7, CP5, CP3, CP1, CPz, CP2, CP4, CP6, TP8, P7, P5, P3, P1, Pz, P2, P4, P6, P8, PO7, PO3, POz, PO4, PO8, O1, Oz, O2, and Iz) positioned according to the 10/10 International System. Additionally, the ground electrode was placed at FPz, the online reference was placed at TP9, and an electrode at TP10 recorded an online signal to “balance” data, in terms of electrode location, in the offline re reference process (see the TMS-EEG session). Horizontal and vertical eye movements were detected by recording electrooculogram (EOG) data. The voltage between the electrode located to the lateral canthus of the left eye and the reference recorded horizontal eye movements. The voltage between the electrode beneath the left eye and the reference, recorded vertical eye movements and blinks. All the electrodes were TMS-compatible Ag/AgCl-coated electrodes mounted on an elastic cap (BrainCap TMS, Brain Products GmbH, Germany). The EEG and EOG signals were bandpass filtered at 0.1–1000 Hz and digitized at a sampling rate of 5 kHz. The impedance was kept below 5 kΩ for all skin/electrode interfaces.

### TMS

Single-pulse TMS was carried out using a biphasic magnetic stimulator connected with a figure-eight coil with a 70-mm diameter (Magstim, Whitland, UK). Each TMS-EEG session consisted of 120 single pulses, for each area, applied at a random interstimulus interval (ISI) of 2–10 s. Target areas were defined as the cortical regions representing the four main nodes for each single-subject DMN (see Characterization of Group and Single-Subject DMNs). The nodes were identified through independent component analysis (ICA) of the individual resting-state fMRI. The coil position and orientation were monitored continuously using a neuronavigation system (SofTaxic, E.M.S., Bologna, Italy) to ensure a high degree of reproducibility across neurophysiological assessments. TMS was applied over the four target areas in blocks in a counterbalanced order. TMS intensity, which was set at 100% of the resting motor threshold (rMT), was defined as the lowest intensity producing motor-evoked potentials with a peak-to-peak amplitude > 50 µV in five out of ten trials in the relaxed first dorsal interosseous muscle of the right hand (Rossini et al. 2015). The hot spot for the motor threshold was found by positioning the coil over the central sulcus and moving it on the scalp in steps of approximately 0.5 cm towards the left motor cortex. Before setting TMS intensity at 100% of the rMT, we evaluated sub- (90%) and supra-threshold (120%) values to investigate the ideal intensity to obtain stable and clear evoked responses in all stimulation conditions. This final setting was done considering: number of TMS pulses, TMS intensity, TEP response, and secondary effects of TMS that would be obtained by raising the stimulation intensity. Moreover, we maintained the same intensity for all stimulation sites (right and left frontal and parietal areas) so that TEPs could be comparable for the stimulation intensity variable. The mean TMS intensity was 66.3 ± 8.8 for the first TMS-EEG session and 66.4 ± 8.2 for the second TMS-EEG session.

## Data preprocessing and statistical analysis

### Structural and functional MRI

Data were preprocessed using SPM12 (http://www.fil.ion.ucl.ac.uk/spm/) running in MATLAB R2017b (The MathWorks, Inc., Natick MA, USA) and MATLAB code developed in-house. The pipeline steps consisted of slice-timing correction, rigid-body realignment, and removal of movement-susceptibility interactions, subtraction of baseline fluctuations by fitting a fourth-order polynomial, low-pass filtering using a second-order Butterworth filter having *f*_−3 dB_ = 0.09 Hz, removal of covariance with the six first-order head movement vectors (translations and rotations) and with average white matter and cerebrospinal fluids derived from individual tissue masks derived from the structural scan. Nuisance regressors were temporally filtered as described above. Head movement magnitude was quantified as median frame-to-frame displacement. Prior to further analyses, the EPI volumes were spatially smoothed through a Gaussian kernel that had a full width at half-maximum value of 8 mm.

### Characterization of group and single-subject DMNs

First, a group ICA using MELODIC (http://fsl.fmrib.ox.ac.uk/fsl/fslwiki/MELODIC) within FSL 5.0.9 (Jenkinson et al. 2012) was performed to extract the group DMN. The DMN was chosen based on a template. To this aim, all subjects’ preprocessed resting-state fMRI data were spatially normalized to the MNI template via linear (affine) registration (Jenkinson et al. 2002), rescaled at a resolution of 4-mm isotropic voxels, and then decomposed into ten independent components (Jovicich et al. 2016) using the multisession temporal concatenation procedure in MELODIC. More components were not extracted to prevent splitting the DMN (Abou-Elseoud et al. 2010; Jovicich et al. 2016). The components and the DMN template were thresholded at z scores > 2.3, *P* < 0.05. Subsequently, a dual regression was used to derive a single-subject DMN from the group DMN (Beckmann et al. 2009; Zuo et al. 2010). Single-subject DMN volume maps were thresholded at z > 2.3, *P* < 0.05. For each subject, the cortical DMN nodes, including the bilateral and symmetric lateral parietal and medial prefrontal cortex, were manually extracted and were the ROIs used to define the structural and functional connectivity measures.

### Diffusion and tractography

DWI data were processed by the concatenation of a step for preprocessing, a step for voxel-based diffusivity model reconstruction, and a step for probabilistic tractography. The analysis pipeline was implemented using FSL and MRtrix (Jenkinson et al. 2012; Tournier et al. 2012). Data were corrected for eddy current distortions and head motion by registering the DW volumes to the first b0 volume. The gradient direction (b-vec) for each volume was corrected by applying individual rotation parameters (Leemans and Jones 2009), and nonbrain voxels were removed with the FSL Brain Extraction Tool (FSL-BET). The fibre orientation distribution function was computed using a constrained spherical deconvolution (CSD) model (Tournier et al. 2007). White- and grey-matter tissues were segmented with FSL using the T1-weighted MRI images associated with each individual brain and then resampled at the resolution of the diffusion MRI data. A final tracking step was carried out using a seed-based probabilistic strategy (Tournier et al. 2010) constrained by the white matter mask. We referred to the ROIs that emerged from the processing and analysis of functional data as seeds for tracking.

This process resulted in an estimate of the most likely pathways connecting each pair of ROIs, namely, the probability of connection measured as the number of streamlines successfully reaching a target voxel from a given seed. The parameter settings used to perform tracking were as follows: step size, 0.5 mm; maximum length, 250 mm; and minimum length, 10 mm. The fibre orientation distribution function (*f*_ODF_) amplitude cut-off was set to 0.1, and for the minimum radius of curvature, we adopted the default value (*90°*  ×  *step size*  ×  *voxel size*). Considering the four ROIs (bilateral lateral parietal and medial prefrontal), the resulting connections corresponded to parts of the corpus callosum called the forceps minor and the forceps major (Fig. 1 left side). The former connects homologous regions of the anterior frontal lobe and the latter connects homologous parieto-occipital regions.

### Structural connectivity measures

After the selection of the connectivity structures of interest, namely, the forceps minor and major (Voineskos et al. 2010), we evaluated some measures that could be used to carry out a quantitative analysis (Yeatman et al. 2012). In the field of tractography, it is possible to define several indexes as measurements of the white matter tracts (Catani et al. 2013; Deslauriers-Gauthier et al. 2019; Mangin et al. 2013). We selected two of the most commonly used indexes in the literature to explore how white matter tracts behaved in correlation with a physiological response.

The first index was the weighted number of fibres, which was defined as the ratio between the number of streamlines between each pair of regions and the total number of streamlines for all seed/target regions, normalized by the number of voxels in each pair to account for differences in the size of seed and target regions (Smith et al. 2015).

The second index was an extracted tensor-derived quantitative measure of the diffusivity metric for each connection, the FA. FA quantifies the directionality of diffusivity in a summative manner (Yeatman et al. 2012).

### TMS-EEG

TMS-EEG data were preprocessed offline (Brain Vision Analyser 2.0 Brain Products GmbH, Munich, Germany). As a first step, cubic interpolation from 1 ms before to 6 ms after the TMS pulse was applied to remove TMS-induced artifacts. Afterwards, a high-pass filter at 1 Hz was applied to the continuous data. Data were then segmented into epochs starting 1250 ms before the TMS pulse and ending 1250 ms after the pulse. The signal was downsampled from 5000 to 1000 Hz. Next, all the epochs were visually inspected, and the EEG epochs containing artifacts or noisy signals were rejected. Physiological and TMS-related artifact components were detected using INFOMAX-ICA and removed based on their scalp distribution, frequency, timing, and amplitude. Subsequently, the low-pass filter at 70 Hz and a notch filter at 50 Hz were applied to the data. All data were rereferenced to the average of all scalp channels, and residual epochs containing artifacts were removed during a second visual inspection. The signal was imported into Fieldtrip (Oostenveld et al. 2011) and resegmented beginning 100 ms before the TMS pulse and ending 400 ms after. Baseline correction was applied using the pre-TMS interval from -100 ms to -1 ms.

### Statistical analysis

First, we aimed to test whether the TEPs recorded in the two TMS-EEG sessions differed in amplitude over space and time. Then, we assessed the Pearson correlation between the TEPs for all the electrodes at each time point, from 7 to 60 ms, with the two indexes of structural connectivity of the forceps, i.e., the weighted number of fibres and FA (i.e., connectivity), in which t tests were run to evaluate the hypothesis that the correlation coefficient R is different from zero. We hypothesized that TEPs of the prefrontal stimulation conditions may be related to indexes in the forceps minor and that TEPs of the parietal stimulation condition may be related to indexes of the forceps major. For these analyses, we conducted nonparametric cluster-based permutation tests to correct for multiple comparisons as implemented by the ft_timelockstatistics function in Fieldtrip (Oostenveld et al. 2011) with MATLAB (2019b, MathWorks).

The comparisons involved all the electrodes for each stimulation condition for the time window of interest from 7 to 60 ms after the TMS pulse. The definition of the time window was based on evidence that the interval is crucial for signal spreading to homologous regions in the opposite hemisphere (Chung et al. 2015; Ilmoniemi et al. 1997; Parks et al. 2012) and to prevent artefacts, i.e., auditory evoked potentials at 100 ms (Conde et al. 2019; Nikouline et al. 1999; ter Braack et al. 2015). Based on the initial one-sample *t* tests, all *t* values above a threshold, corresponding to an uncorrected *p* value of 0.05, were grouped into clusters based on adjacent significant time points and electrodes, considered separately, for a sample with positive and negative *t* values (two-tailed test). For a significant sample to be included in a cluster, it was required to have at least one adjacent neighbouring significant sample in both space (electrodes) and time. The spatial neighbourhood of each electrode was defined as all electrodes within approximately 5 cm, resulting in a mean of 6.3 (min = 3, max = 8) and median of 7 neighbours per electrode. The t values within each cluster were then summed to produce a cluster statistic. Subsequently, this procedure was repeated across 2500 permutations by calculating Monte Carlo estimates of the significance probabilities (*p* < 0.05).

## Results

### TMS-EEG data comparison

The statistical comparison performed on all electrodes in the time window from 7 to 60 ms after the TMS pulse did not reveal any spatiotemporal differences in the two TMS-EEG sessions (all ps > 0.05), as reported in S1. Due to this result, data from the two sessions were merged before the correlations with the structural connectivity measures were evaluated.

### TMS-EEG and structural measure correlations

**Fig. 2:**
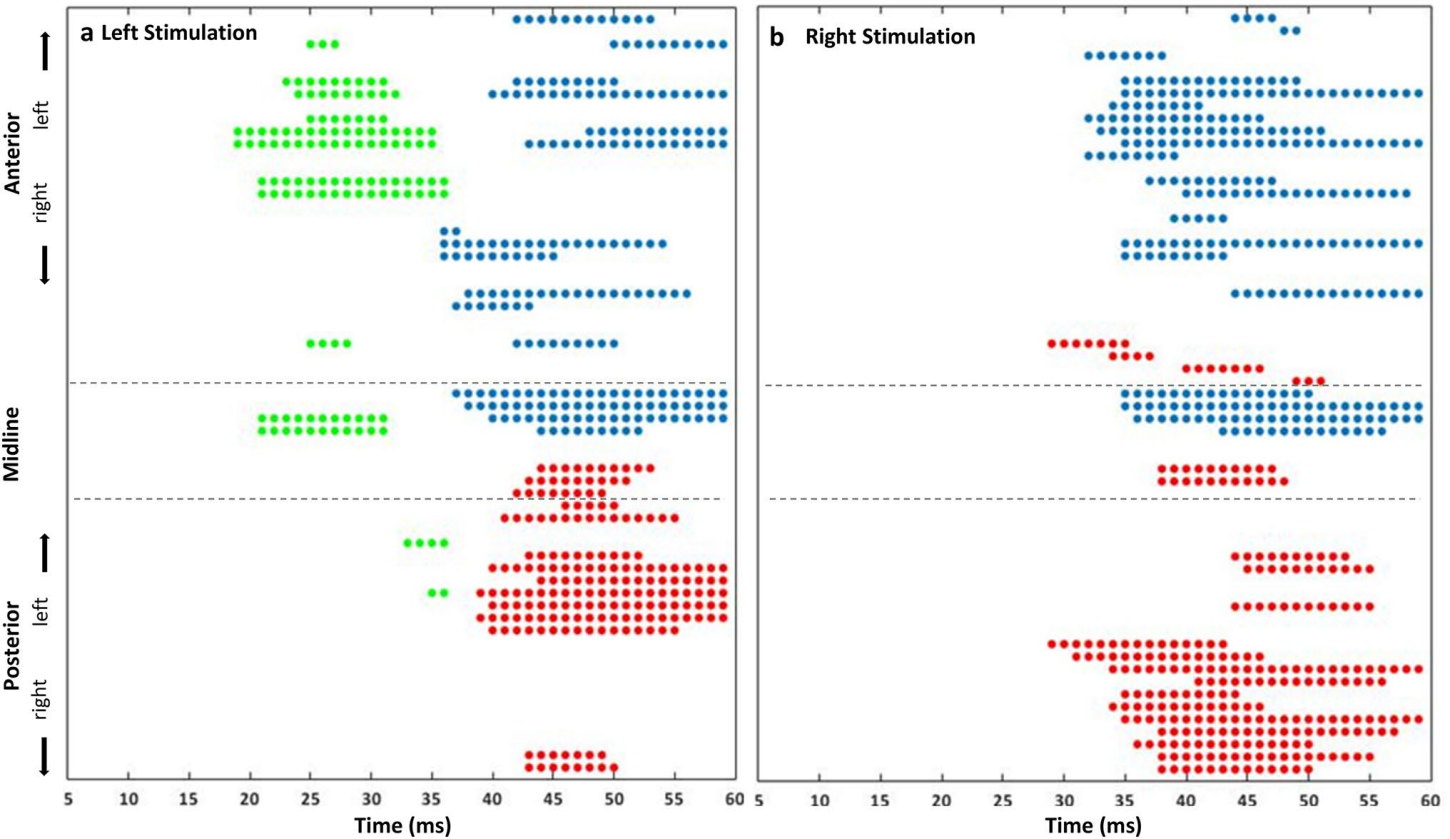
Significant cluster distribution of electrodes and time (distinct colours for each cluster). In the left panel (**a**), the clusters for the left parietal stimulation condition are depicted. In the right panel, (**b**) the clusters for the right parietal stimulation condition are shown. The blue clusters are positively correlated, while the red and green clusters are negatively correlated

The statistical analysis performed between the TEPs and the FA values of the forceps major revealed significant correlations for the left (Fig. 2a) and right (Fig. 2b) parietal stimulation conditions, with a similar pattern of results, as described in the following section. Instead, the correlation between the TEPs and the FA values of the forceps minor was not significant (all ps > 0.05). Similarly, statistics did not reveal any significant correlation between TEPs and the weighted number of fibres in any stimulation conditions (all ps > 0.05).

The interpretation of the clusters of correlations depended on the voltage of the signal with which it was correlated. If the voltage was positive, a positive cluster indicated that the signal increased with increasing FA, and a negative cluster indicated that the signal decreased with increasing FA. On the other hand, if the voltage was negative, a positive cluster indicated that the signal decreased with increasing FA, and a negative cluster indicated that the signal increased with increasing FA. The results described below reflect these relationships.

### Left parietal TEPs and FA correlation

**Fig. 3:**
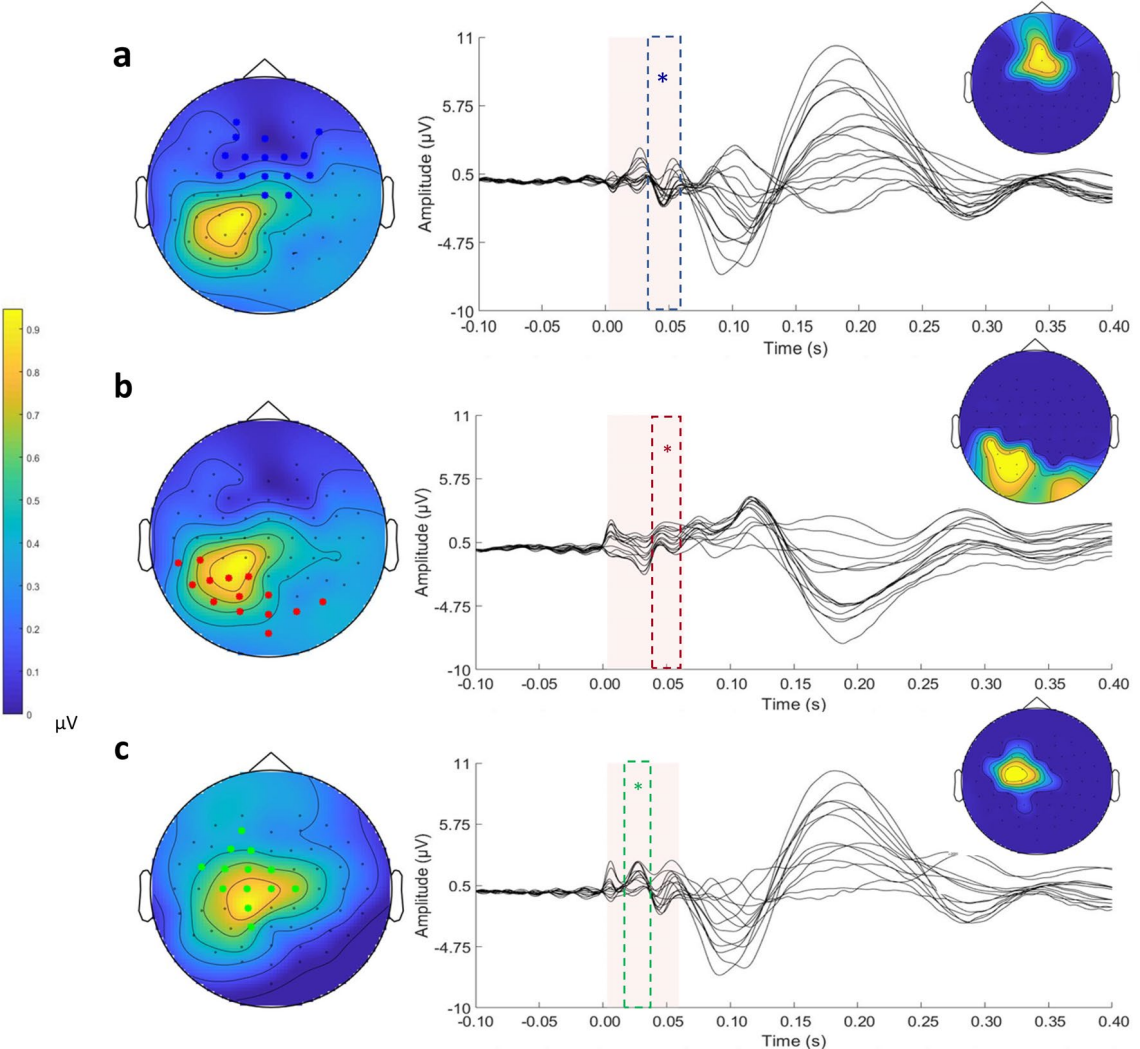
Significant clusters of correlation for the left parietal stimulation condition, one positive (**a**), and two negative (**b** and **c**), are shown. For each cluster, the butterfly plot of the electrodes that are part of the cluster is displayed. Dashed lines indicate the temporal extension of each significant cluster. The topographies on the left indicate the voltage of the signal over all electrodes for the significant time window and indicate the electrodes that are part of the cluster. The topographies on the right indicate the scalp distribution of the cluster R values. The blue electrodes are part of the positive cluster, while the red and green electrodes are part of the negative cluster

The correlations revealed three significant clusters (Fig. 3); one was positive (*p* = 0.002) and two were negative (*p* = 0.022; *p* = 0.045).

The positive cluster included the signal from frontocentral electrodes (FP1, AF3, AFz, AF8, F3, F1, Fz, F2, F4, FC3, FC1, FCz, FC2, FC4, Cz, and C2) in the time interval of 37 to 60 ms (Fig. 3a and S2a). Considering that the signal of this cluster was negative on average, as shown in the topography in Fig. 3a, this result indicated that the TEP amplitude in frontocentral sites decreased for higher values of FA in the forceps major.

The first negative cluster included parieto-occipital electrodes (TP7, CP5, P7, P5, P3, P1, PO7, PO3, POz, O1, O2, Oz, Iz, and PO8) on both hemispheres in the interval of 40 to 60 ms (Fig. 3b and S2b). When the TEP voltage was positive, as was the case for the electrodes close to stimulation, this correlation indicated that the TEP amplitude decreased for higher FA in the forceps major. In contrast, when the TEP voltage was negative, as was the case for the electrodes of the contralateral hemisphere, this correlation indicated an increase in the TEP amplitude.

Finally, the second negative cluster comprised left frontocentral electrodes (AF3, F3, F1, FC5, FC3, FC1, FCz, C3, C1, Cz, C2, CP1, and P1) in the time interval of 20 to 37 ms (Fig. 3c and S2c). As shown in Fig. 3c, the voltage on these electrodes and in this time window was positive. Therefore, this correlation indicated a decrease in signal amplitude with higher FA values of the forceps major.

The pattern that emerged from the significant clusters demonstrated a general reduction in the voltage recorded from electrodes close to the stimulation site and over frontocentral areas and an increase in the voltage in posterior electrodes contralateral to the TMS target in correlation with the FA values of the forceps major.

### Right parietal TEPs and FA correlation

**Fig. 4:**
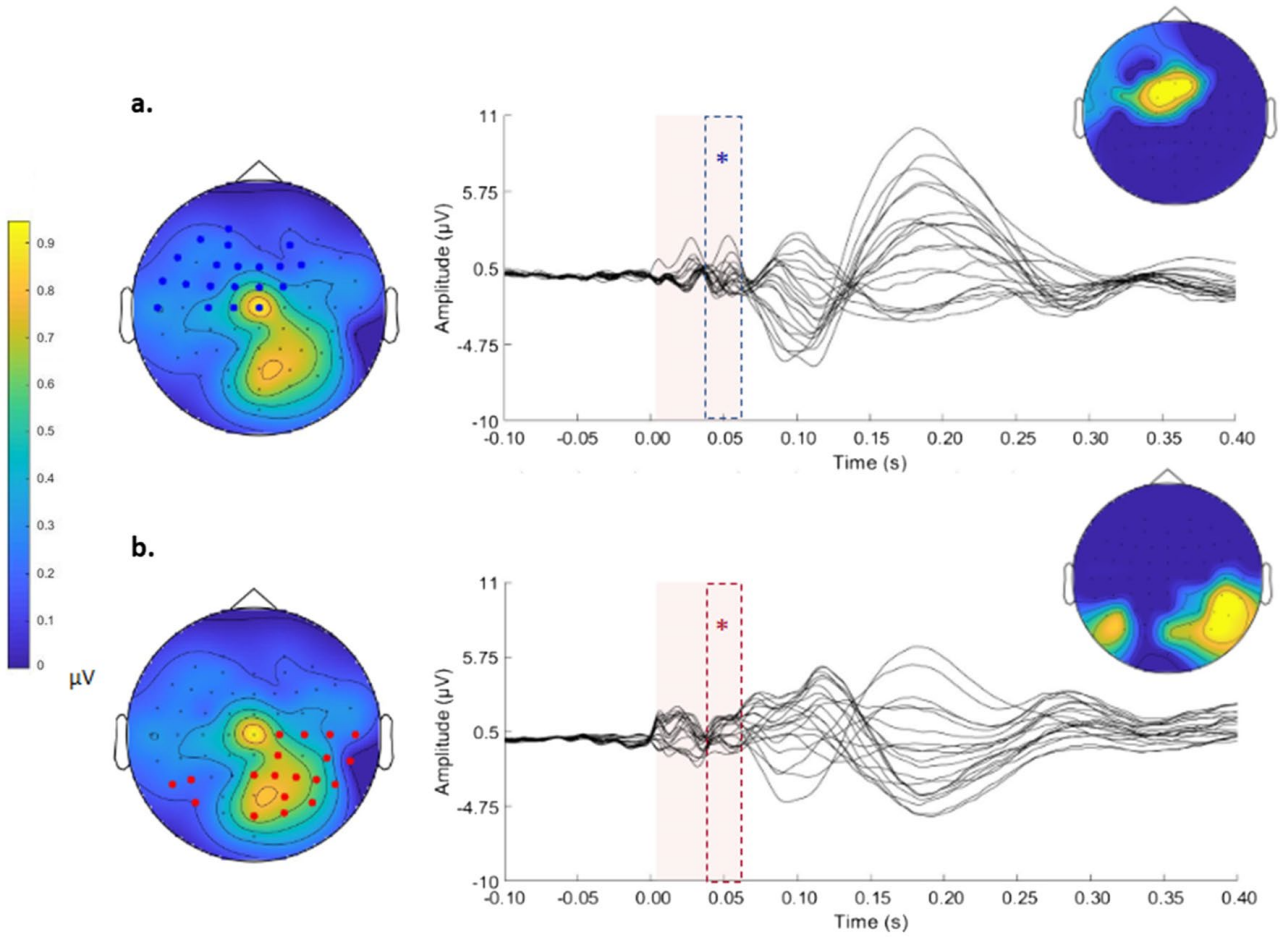
Significant clusters for the right parietal stimulation condition, one positive (**a**) and one negative (**b**), are shown. For each cluster, the butterfly plot of the electrodes that are part of the cluster is displayed. Dashed lines indicate the temporal extension of each significant cluster. The topographies on the left show the voltage of the signal over all electrodes for the entire significant time window and the electrodes that are part of the cluster. The topographies on the right show the distribution of the cluster statistics. The blue electrodes are part of the positive cluster, and the red electrodes are part of the negative cluster

Statistical analysis revealed two significant clusters (Fig. 4), one of which was positive (*p* = 0.003) and one of which was negative (*p* = 0.007), with a pattern similar to that observed with left parietal stimulation.

The positive cluster involved frontocentral electrodes (FP1, AF7, AF4, AFz, F7, F3, F1, Fz, F2, F4, FT7, FC5, FC3, FC1, FCz, FC2, T7, Cz, C3, and C1) for the time interval of 33 to 60 ms (Fig. 4a and S3a). Considering that the signal of this cluster was negative on average, as shown in the topography in Fig. 4a, this result indicated that the TEP amplitude in frontocentral sites decreased for higher values of FA in the forceps major.

The negative cluster included parieto-occipital electrodes of both hemispheres (C2, C4, C6, T8, CP2, CP4, CP6, TP8, P2, P4, P6, P8, PO8, PO4, Pz, O2, Oz, PO7, P7, and P5) for the interval of 30 to 60 ms (Fig. 4b and S3b), in which voltage was positive, on average, in electrodes near the stimulation site and negative, on average, in the contralateral site. Therefore, this correlation indicated a decrease in signal amplitude for the stimulation site and an increase in amplitude for the contralateral site.

For the left parietal condition, considering the frontocentral electrodes (positive cluster), the negative voltage decreased with higher FA. In the bilateral parieto-occipital electrodes (negative cluster), the positive voltage on electrodes ipsilateral to stimulation decreased as FA increased. Conversely, voltage on electrodes contralateral to stimulation increased with higher FA values for the forceps major.

## Discussion

In this study, our principal aim was to explore the relationship between the structural and functional characteristics of DMN connectivity (Momi et al. 2021). Our results demonstrated a link between the neurophysiological response of the DMN and the underlying anatomical connections. The reported results were derived from a methodologically integrated approach, with which we obtained a dynamic view of DMN connectivity.

To investigate the relationship between the physiological indexes and the underlying structural connections, the correlation between the TEPs and the indexes of structural connectivity of the two forceps was assessed. We found that there were correlations between TEPs and the FA values of the forceps major for the left (Figs. 2a and 3) and right (Figs. 2b and 4) parietal stimulation conditions. Therefore, stimulating one of the two bilateral parietal nodes induced a pattern of activity that correlated with the forceps major FA.

First, the white matter tract diffusivity of the forceps major correlated with the activity in areas that were connected by it, i.e., parietal areas (Figs. 3b and 4b). Accordingly, both left and right parietal stimulation induced a negative cluster with positive voltage over parieto-occipital electrodes that included both ipsilateral and contralateral electrodes (~ 40–60 ms). This cluster indicated that the positive voltage signal over ipsilateral electrodes decreased when FA values of the forceps major were higher, and that the negative voltage signal over contralateral electrodes increased when FA values of the forceps major were higher.

Interestingly, the white matter tract diffusivity of the forceps major also correlated with activity detected by frontal electrodes (Figs. 3a and 4a). Both left and right parietal stimulation induced a positive cluster with negative voltage (~ 40–60 ms) over frontocentral electrodes, so that the higher the FA in the forceps major was, the smaller the TEP signal was. Moreover, left parietal stimulation induced a negative early cluster with positive voltage (~ 30 ms) over the left frontocentral electrodes; thus, the higher FA values of the forceps were, the smaller the TEP signal was (Fig. 3c).

Therefore, even if TEPs have been correlated with a specific fibre tract underlying the connectivity of the parietal nodes, the induced response that showed an association was widely distributed and included both parietal and frontocentral electrodes.

The spatial location of these clusters corresponds to the principal cortical nodes of the DMN, suggesting a possible link. As described in the EEG literature, these electrodes are relevant in the detection of DMN activity (Chang et al. 2013; Laufs et al. 2003; Mulert, 2013; Neuner et al. 2014; Nunez and Silberstein, 2000). Moreover, they overlapped with the fMRI map of the DMN for each subject.

Our results may be described as temporal dynamics of the DMN, within its spatial organization, which corresponds to the necessary integration for obtaining efficient processing of neural information (de Pasquale et al. 2018, Momi et al. 2020). Previous work has demonstrated that two major features guide integration: the brain network structural topology and the functional connectivity dynamics; in addition, hub regions regulate “network traffic” (from de Pasquale et al. 2018). The cortical nodes of the DMN have a crucial role in the functional organization of the network, which corresponds to a structural architecture that supports this organization. The TMS pulse is able to induce energy, detected via EEG, which dynamically diffuses within the main functional nodes of the network through its related structural pathways. Notably, our result underlies a different role that the two parietal nodes have with respect to the component recorded in the frontocentral left-lateralized region. A different role for the two hemispheres in modulating attention through corpus callosum connections is a likely explanation of these results (Corbetta and Shulman 2011; Koch et al. 2011; Palmer et al. 2013).

A possible reason why the correlations were significant only for the parietal stimulation conditions might be the topological configuration of the prefrontal nodes. The bilateral prefrontal nodes are in a medial position, so they are geometrically close to each other (Lee et al. 2018). Therefore, considering the stimulation protocol, in these conditions, it might be challenging to measure a TEP that correlates with the structural pathway. The coil location, for both prefrontal nodes, may result in the electrodes near the stimulation site being more sensitive to the currents induced by the TMS pulse and a consequent spread of current, making it difficult to detect the distribution of the neurophysiological responses. While, the bilateral symmetric parietal nodes are more distant from each other than the prefrontal nodes. The reported results support the notion that the proposed approach is feasible to use to investigate the relation between the structural connectivity and the neurophysiological responses of the DMN. The correlational outcomes can provide more insights into the behaviour of the neurophysiological measures recorded with TMS-EEG with respect to the underlying structural pathways (Levy-Lamdan et al. 2020). The results showed an extended relation of every single node within networks since the stimulation of each of the cortical nodes of the DMN correlated with its underlying pathway and returned a dynamically integrated outcome. This implied that the diffusion of information occurs efficiently through the functionally connected nodes and accounts for the impact of direct structural connections.

Our results regarding DMN connectivity were acquired through an approach that has not previously been used, the combination of MRI, TMS, and EEG, to obtain the spatiotemporal relation of DMN connectivity. This approach has been challenging, mainly because we had no previous evidence to guide us when initially defining the best options for all the measurements. The present results provided evidence that it is feasible to combine different methods for exploring DMN connectivity, thus offering more tools that can be used to explore brain function (Momi et al. 2021).

However, we faced some difficulties that created limitations in this study. The first challenge faced was the need to find conjunctions within all the different neuroimaging methods. The spatiotemporal characteristics of each involved method were different and thus led us to focus on cortical information. This is an important limitation because the DMN is composed of both cortical and subcortical regions. In this study, using TMS-EEG, we were not able to consider the role and the influence of deeper brain regions. Of course, the inability to incorporate these aspects makes it difficult to achieve a deeper comprehension of dynamic integration based on brain network processing. Another consequence of this issue is the restriction of the reconstruction of white matter pathways. We used the cortical nodes that emerged from the functional MRI data of each subject that were in the cortical region, quite far from white matter. A further limitation of this study might be the temporal distance between the resting-state fMRI and the TMS-EEG measurements. Since the reproducibility of the resting-state networks decreases as the time between the test and retest scan increases, this issue could have potentially biased our results (Noble et al 2019). However, the results of the meta-analysis reported in the abovementioned study suggested that the DMN is the most stable network that can be investigated over time by resting-state fMRI.

Most likely, if we were able to obtain more in-depth ROIs, more tracts would emerge, and many more underlying pathways would be revealed. More evidence is needed to study the measurements that can be obtained from the integrative approach, how measurements can replicate our results, and how these results can be utilized. Future directions should explore how, for instance, other structural connectivity indexes can replicate the comparison outcomes of this study and should investigate whether higher-order analyses, such as time–frequency and source analyses, can describe the dynamical interaction.

In conclusion, this study provided evidence of the potential of an integrative approach for exploring the integrated organization of the DMN. The correlations between the neurophysiological data and the underlying pathways have demonstrated the possibility of investigating connectivity dynamically in a comprehensive scenario of the brain at rest. A significant advantage of this method highlighted by this study is that the correlation between the temporal dynamics and the underlying spatial pathways reverberates within the cortical nodes of the network. We hypothesize that this dynamic integration reflects the information processing that is visibly dependent on the temporal scales of connectivity across the brain. The proposed approach represents a promising technique for investigating brain connectivity in both healthy individuals and those with pathological conditions.

## Supplementary Information

Below is the link to the electronic supplementary material.

Supplementary file1 (DOCX 464 KB)
