# Supplementary material for: An integrated TMS-EEG and MRI approach to explore the interregional connectivity of the default mode network
Source: Brain Struct Funct. 2022 Feb 4;227(3):1133–44. doi: 10.1007/s00429-022-02453-6 (PMC8930884; doi:10.1007/s00429-022-02453-6)

**Supplementary Statistical Analysis and Results**

We tested the correlations between the TEPs for prefrontal targets in all electrodes and in a time frame of 7 to 60 ms, with FA values of the forceps major, and, similarly, the correlations between TEPs for parietal targets and FA values of the forceps minor. Moreover, the correlation between the FA values of the two forceps was evaluated. We hypothesized that if the frontal and posterior tracts were correlated, the TEPs of the prefrontal stimulation conditions may be related to indexes in the forceps major, and TEPs of the parietal stimulation condition may be related to indexes of the forceps minor. For these analyses, we conducted nonparametric cluster-based permutation tests to correct for multiple comparisons as developed in the ft_timelockstatistics function in Fieldtrip (Oostenveld et al. 2011) with MATLAB (2019b, MathWorks) and a Spearman correlation with STATISTICA (StatSoft, Inc. (2007) (data analysis software system, version 8.0. [www.statsoft.com](http://www.statsoft.com).). Only significant correlations are reported in the results.

## *Correlation Between Left Parietal TEPs and FA of the Forceps Minor*

Statistical analysis revealed two significant clusters, one positive (p = 0.0304) and one negative (p = 0.0072), with a scalp distribution among the left parietal and bilateral prefrontal electrodes (Figure S4a).

The positive cluster (Figure S4b) involved bilateral frontocentral electrodes (F3, F1, Fz, F2, FC1, FCz, FC2, FC4, FC6, C2, C4, C6, CP2, and CP4) for the time interval of 39 to 60 ms (Figure S4). Considering that the signal of this cluster was positive on average, this result indicated that TEP amplitude in frontocentral sites increased with higher values of FA in the forceps minor.

The negative cluster (Figure S4c) included frontoparietal electrodes of both hemispheres (F3, F1, Fz, FC3, FC1, FCz, FC2, C3, C1, Cz, C2, CP5, CP3, CP1, CPz, P5, P3, P1, PO7, PO3, O1, and Iz) for the interval of 19 to 60 ms (Figures S4), in which voltage was positive on average. Therefore, this correlation indicated a decrease in signal amplitude for the stimulation site and for the frontal ipsilateral site.

## *Correlation Between Right Frontal TEPs and FA of the Forceps Major*

Statistical analysis revealed one significant positive (p = 0.011) and one negative (p = 0.047) cluster (Figure S5a) with a scalp distribution among the bilateral fronto-parietal electrodes.

The positive cluster (Figure S5b) involved bilateral frontocentral electrodes (FP2, AFz, AF4, AF8, F3, F1, Fz, F2, F6, FC3, FC1, FCz, FC2, FC4 and C2) for the time interval of 31 to 60 ms. Considering that the signal of this cluster was positive on average, this result indicated that TEP amplitude in frontocentral sites increased for higher values of FA in the forceps major.

The negative cluster (Figure S5c) involved bilateral fronto-parietal electrodes (F7, FT7, T7, TP7, P7, P5, P3, P6, P8, PO7, PO3, POz, PO4, PO8 and O1 ) for the time interval of 38 to 60 ms. Considering that the signal of this cluster was positive on average, this result indicated that TEP amplitude in fronto-parietal sites decreased for higher values of FA in the forceps major.

*Correlation Between the Forceps Minor and Forceps Major*

We assessed the relation between FA values of the two tracts with a Spearman correlation. The two tracts were shown to be positively correlated (Spearman R = 0.368, p = 0.038).

**Supplementary Figure** **Captions**

**Figure S1** TEPs recorded in the electrodes underlying the TMS coil per stimulation condition, test, and retest are plotted (blue = 1^st^ and red = 2^nd^ TMS-EEG acquisition session). No significant results were observed.


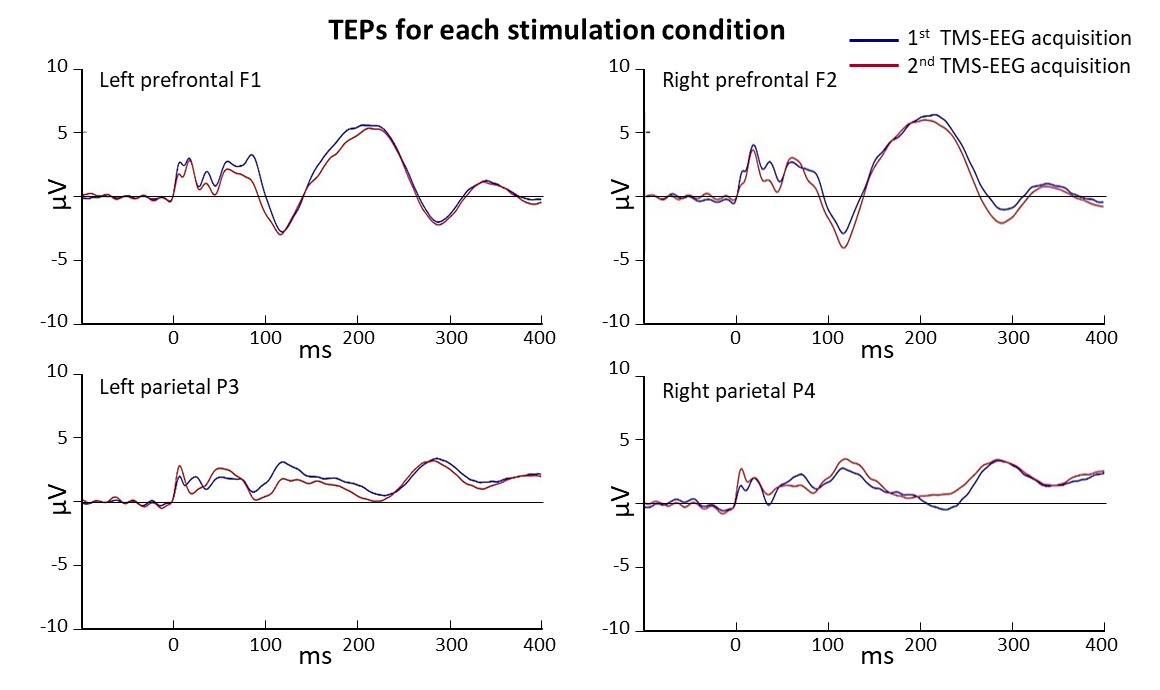
**Figure S2** The first row represents the values of the cluster statistics for each significant cluster of the left parietal stimulation condition. In the second row, the spatiotemporal distribution of the clusters is reported. The first column (a) corresponds to the positive cluster, while b and c show the two negative clusters.

**
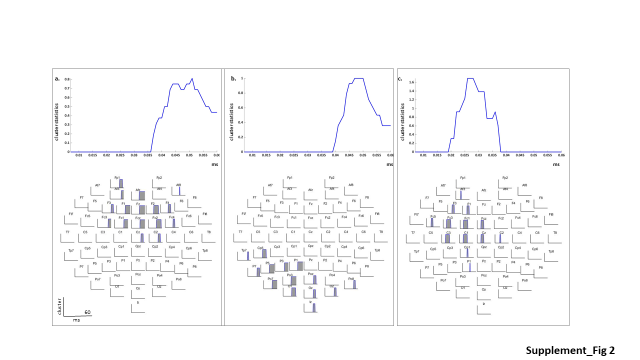
**

**Figure S3** The first row shows the values of the cluster statistics for each significant cluster of the right parietal stimulation condition. The spatiotemporal distribution of the clusters is reported in the second row. The first column (a) corresponds to the positive cluster, and b represents the negative cluster.

**
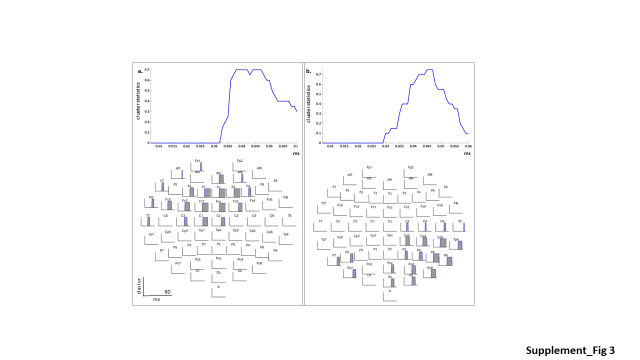
**

**Figure S4** The first column (a) represents the TEP amplitude in the time window of 0 to 60 ms of the left parietal stimulation condition, overlaid with the significant clusters emerging from the correlation with the FA values of the forceps minor. Panels b and c depict a plot of the positive and negative significant clusters, respectively.


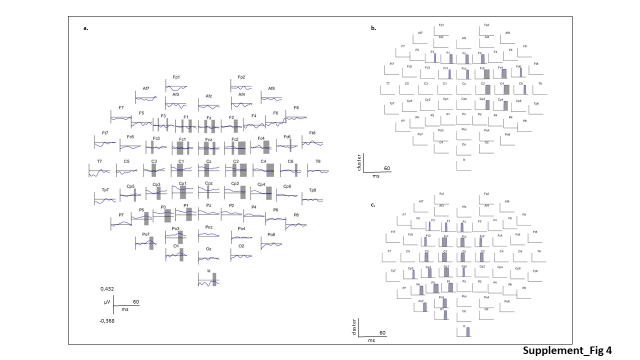


**Figure S5** The first column (a) represents the TEP amplitude in the time window of 0 to 60 ms of the right prefrontal stimulation condition, overlaid with the significant clusters emerging from the correlation with the FA values of the forceps major. Panel b and c shows a plot of the data for the positive and negative significant clusters, respectively.


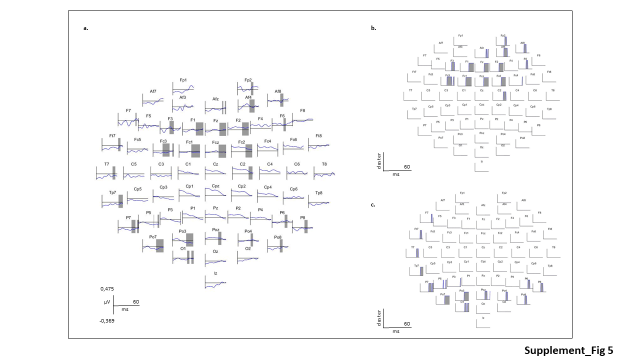

Supplement: Supplementary file 1 — Supplementary file1 (DOCX 464 KB) [file 429_2022_2453_MOESM1_ESM.docx]
